# Rapid and Durable Response With Nab-Sirolimus After Everolimus Failure in a Patient With Perivascular Epithelioid Cell Tumors (PEComas) of the Uterus

**DOI:** 10.7759/cureus.14951

**Published:** 2021-05-11

**Authors:** Pallavi Kopparthy, Martina Murphy

**Affiliations:** 1 Medicine, University of Florida, Gainesville, USA; 2 Hematology and Oncology, University of Florida Health, Gainesville, USA

**Keywords:** gynecological cancer, pharmacology and therapeutics, pecoma

## Abstract

Perivascular epithelioid cell tumors (PEComas) are rare mesenchymal tumors with a natural history ranging from indolent benign lesions to ones with an aggressive clinical course including distant metastases. Recent reports have suggested that mTOR inhibitor sirolimus and related drugs show some benefit in non-tuberous sclerosis complex PEComas. However, therapeutic options for patients who progress on sirolimus are very limited. We describe a patient with metastatic uterine PEComa, who progressed on mTOR inhibitor everolimus but had a rapid and durable response to nab-sirolimus.

## Introduction

Perivascular epithelioid cell tumors (PEComas) are rare mesenchymal tumors that show immunophenotypic features of smooth muscle and melanocytic differentiation. The term PEComa was first used in 1996 to designate neoplasms containing epithelioid cells with a close association to vessel walls and recently recognized as a distinct entity by the World Health Organization [1]. They include tumors like renal angiomyolipoma, pulmonary lymphangioleiomyomatosis (LAM), which are relatively benign lesions and seen in patients with tuberous sclerosis complex (TSC). However, the spectrum also includes rarer tumors of variable malignant potential involving lung, gynecologic and gastrointestinal systems; the latter not common in TSC [2].

Evidence from a registration trial in advanced malignant PEComa (AMPECT) suggests that patients with AMPECT with no prior exposure to mTOR inhibitors, particularly those with TSC1 or TSC2 mutations, benefit from the use of an investigational mTOR inhibitor, nab-sirolimus (also known as albumin-bound sirolimus nanoparticles and ABI-009) [3]. An expanded access protocol (EAP) bridging between the closure of the AMPECT study and the marketing approval of the product gives patients with serious condition access to treatment with nab-sirolimus. AMPECT patients with and without prior exposure to mTOR inhibitors, other than nab-sirolimus were included. Here we report a case of metastatic uterine PEComa enrolled in the EAP, who progressed on the mTOR inhibitor everolimus yet had a rapid and durable response to nab-sirolimus.

## Case presentation

Diagnosis

A 58-year-old post-menopausal female presented with abnormal uterine bleeding. Endometrial biopsy revealed a neoplastic process and further workup with a CT scan showed a 7cm mass in the uterus. No other metastatic lesions were found. Following this, a laparoscopic hysterectomy with bilateral salpingo-oophorectomy was performed and pathology was consistent with malignant PEComa, which stained positive for smooth muscle actin, HMB-45, and Melan-A (59 mitoses per 10hpf). Foundation one genomic testing was positive for TSC1 with stable microsatellite status and low tumor mutation burden.

Treatment history

Chemotherapy

**Figure 1:**
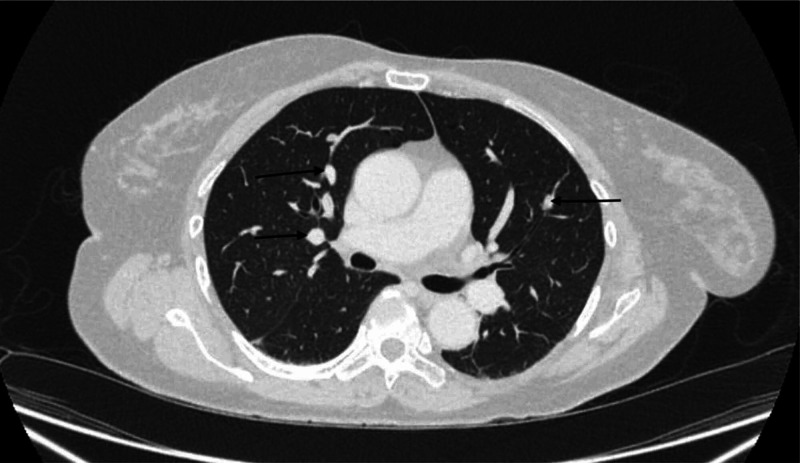
Computed tomography of chest showing multiple pulmonary nodules (black arrows) prior to starting everolimus 10mg PO.

The primary tumor was locally advanced at diagnosis, adjuvant chemotherapy was not administered, and the patient was monitored with serial scans. CT scan at six months following surgery showed multiple bilateral pulmonary nodules, consistent with metastatic disease (Figure 1).

mTOR Inhibitor, Everolimus

**Figure 2:**
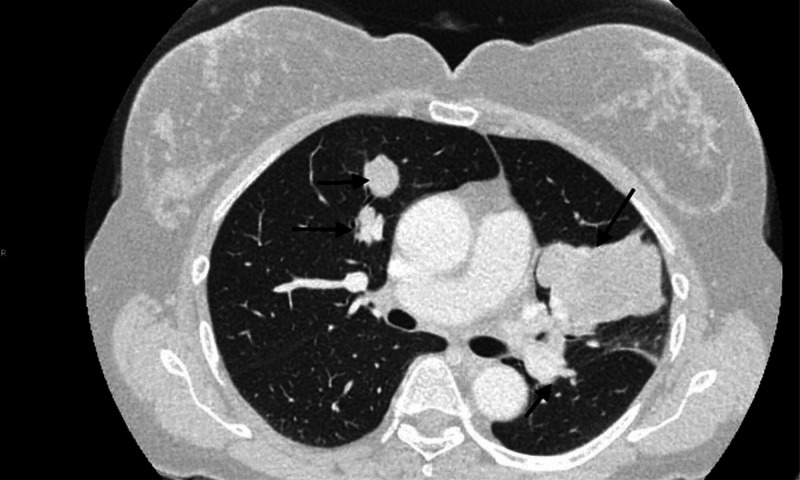
Computed tomography of the chest showing a significant progression of disease in lungs (black arrow) two months after starting everolimus and prior to starting nab-sirolimus.

With the finding of metastatic disease, the patient was started on 10mg everolimus PO daily. Three weeks after treatment, the patient was hospitalized due to fever and headache, related to everolimus, and the dose was reduced to 5mg PO daily which was gradually uptitrated to 5mg daily in four weeks. CT scan two months after starting everolimus demonstrated marked interval enlargement of all pulmonary lesions seen on prior imaging, along with new lesions, indicative of progressive disease (Figure 2). Additionally, brain imaging performed for evaluation of dizziness showed a new enhancing lesion in the periphery of the left occipital lobe. Prior scans were negative for any intracranial lesion.

Investigational mTOR Inhibitor, Nab-Sirolimus

**Figure 3:**
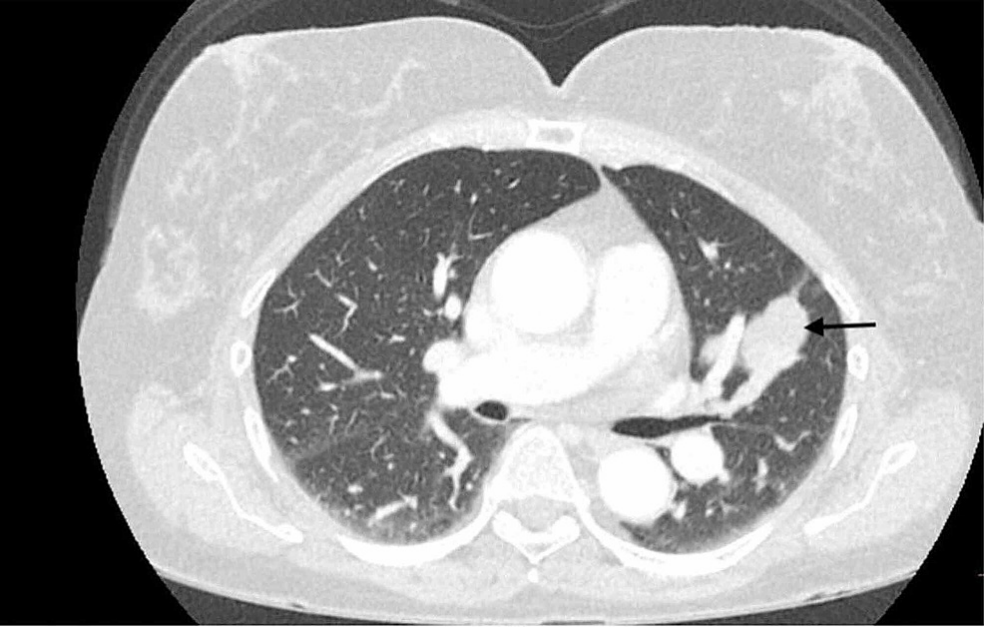
Computed tomography of chest showing a decrease in size of pulmonary nodules (black arrow) three months after starting nab-sirolimus.

After the failure of treatment with everolimus, the patient was enrolled in an expanded access protocol, PEX-002, with nab-sirolimus at 100 mg/m^2^ on day 1 and day 8 of a 21-day cycle. She also received stereotactic radiosurgery to the metastatic lesion in her brain. The six-week restaging following two cycles of therapy showed a marked decrease (50%) in the target tumor lesion in her chest, indicating partial response, which was confirmed by the week 12 scans (Figure 3). The MRI brain also showed a reduction in the size of the cranial lesions.

Clinical symptoms prior to nab-sirolimus included coughing-up blood, which ceased after two cycles, enabling her to run two miles without “getting winded.” The patient developed grade 2 thrombocytopenia after cycle 2 for which the dose was reduced to 75mg/m^2^. Other treatment-related adverse events were elevated lipids, maculopapular rash (grade 2), which were managed appropriately.

## Discussion

PEComa is a rare form of sarcoma and has no effective therapy in the metastatic setting leading to a fatal outcome in most cases. Our patient has uterine PEComa, which is the most common site for PEComa’s not associated with TSC and commonly behave as a high-grade sarcoma with distant metastases and death [4]. She also had a clinically aggressive disease based on size, >1mitoses/50 high power field.

Mutations in cytoplasmic tumor suppressor genes TSC1 or TSC2 are commonly seen in TSC [5]. Gene products of TSC1, TSC2 negatively regulates mTORC1 through inhibition of the mTOR kinase activator RHEB GTPase [6]. Thus, the absence of normally functioning TSC1/2 leads to increase mTOR activity, causing tumors to develop in various organ systems including the kidney, lung, brain and skin. Limited studies have demonstrated a similar mechanism of mTOR hyperactivation in non-TSC PEComas [7,8]. Cases published by Wagner et al., Italiano et al., Subbaiah et al. and Dickson et al. strongly support this model and demonstrate the use of mTOR inhibitors as a rational therapeutic target in patients with non-TSC PEComas. Some PEComa’s are resistant to mTOR inhibitor treatment, despite optimal dosing and treatment as seen in our case. A few PEComas have a unique alveolar epithelioid rearrangement that harbor a TFE3 mutation and while these tumors have a normal expression of TSC2 they do not have TSC2 LOH [9]; however, this was not demonstrated in our patient and does not explain the cause for mTOR inhibitor resistance in all PEComa’s and definitely is an area of future research. A combination of genetic findings in TSC1/TSC2 and staining for pS6-S235/236 may predict response to sirolimus/everolimus but we do not have enough data to support this.

The intriguing aspect about our patient is that even though she progressed through everolimus, she had a durable response to nab-sirolimus an albumin-bound sirolimus nanoparticle, also known as ABI-009 or nab-rapamycin, an intravenous mTOR inhibitor. nab-Sirolimus utilizes an established nanoparticle albumin-bound technology, resulting in a distinct PK profile, significantly higher anti-tumor activity, intra-tumoral drug accumulation, and mTOR target suppression [10,11]. This compound received Breakthrough Therapy Designation from the US Food and Drug Administration (FDA) in December 2018, for the treatment of patients with advanced (metastatic or locally advanced) malignant PEComa based on the AMPECT trial; these patients had no prior exposure to mTOR inhibitor. To our knowledge, this is the first patient who responded to nab-sirolimus after progressing through mTOR inhibitor.

## Conclusions

Cytotoxic chemotherapies for sarcoma show minimal benefit in PEComas and there are currently no drugs approved for this disease. Malignant PEComas frequently harbor mutations in the TSC1 and/or TSC2 genes that result in the activation of mTOR pathway. Therefore, mTOR signaling is a promising therapeutic target for malignant PEComa. NabSirolimus may therefore provide an important therapeutic option for these patients with locally invasive and metastatic PEComa beyond the currently available treatments and further research is definitely required in this area.
